# Moving Forward in Human Cancer Risk Assessment

**DOI:** 10.1289/ehp.1002735

**Published:** 2010-12-13

**Authors:** Richard S. Paules, Jiri Aubrecht, Raffaella Corvi, Bernward Garthoff, Jos C. Kleinjans

**Affiliations:** 1 National Institute of Environmental Health Sciences, National Institutes of Health, Department of Health and Human Services, Research Triangle Park, North Carolina, USA; 2 Pfizer Inc., Groton, Connecticut, USA; 3 In vitro Methods Unit/European Centre for the Validation of Alternative Methods, Institute for Health and Consumer Protection, European Commission Joint Research Centre, Ispra, Italy; 4 BIO.NRW Cluster Biotechnologie North Rhine–Westphalia, Düsseldorf, Germany; 5 Maastricht University, Maastricht, the Netherlands

**Keywords:** cancer, human, omics technologies, risk assessment, systems biology

## Abstract

**Background:**

The current safety paradigm for assessing carcinogenic properties of drugs, cosmetics, industrial chemicals, and environmental exposures relies mainly on *in vitro* genotoxicity testing followed by 2-year rodent bioassays. This testing battery is extremely sensitive but has low specificity. Furthermore, rodent bioassays are associated with high costs, high animal burden, and limited predictive value for human risks.

**Objectives:**

We provide a response to a growing appeal for a paradigm change in human cancer risk assessment.

**Methods:**

To facilitate development of a road map for this needed paradigm change in carcinogenicity testing, a workshop titled “Genomics in Cancer Risk Assessment” brought together toxicologists from academia and industry and government regulators and risk assessors from the United States and the European Union. Participants discussed the state-of-the-art in developing alternative testing strategies for carcinogenicity, with emphasis on potential contributions from omics technologies.

**Results and Conclusions:**

The goal of human risk assessment is to decide whether a given exposure to an agent is acceptable to human health and to provide risk management measures based on evaluating and predicting the effects of exposures on human health. Although exciting progress is being made using genomics approaches, a new paradigm that uses these methods and human material when possible would provide mechanistic insights that may inform new predictive approaches (e.g., *in vitro* assays) and facilitate the development of genomics-derived biomarkers. Regulators appear to be willing to accept such approaches where use is clearly defined, evidence is strong, and approaches are qualified for regulatory use.

In a lecture at the “Genomics in Cancer Risk Assessment” workshop in 2009, Hans Lehrach of the Max Planck Institute for Molecular Genetics stated that, “Life is the translation of the information in the genome into the phenotype of the organism. The organism ‘computes’ this phenotype from its genotype, given a specific environment.” The goal of human risk assessment is to decide whether a given level of exposure to a particular chemical or substance is acceptable to human health and to provide risk management measures based on an evaluation and prediction of the effects of that exposure on human health. Within this framework, the current safety paradigm for assessing possible carcinogenic properties of drugs, cosmetics, industrial chemicals, and environmental exposures relies mainly on *in vitro* genotoxicity testing followed by 2-year bioassays in mice and rats. This testing paradigm was developed 40–50 years ago with the initial premise that “mutagens are also carcinogens” (Ames 1973) and that the carcinogenic risk to humans can be extrapolated from the tumor incidence in rodents after lifetime exposure to maximally tolerated doses of chemicals (Weisburger 1983).

Genotoxicity testing is used as a surrogate for carcinogenicity testing and is required for initiation of clinical trials (Jacobs and Jacobson-Kram 2004) and for most safety assessments of industrial chemicals. Although the carcinogenicity-testing paradigm has effectively protected patients and consumers from the introduction of harmful carcinogens as drugs and other products, the testing paradigm is clearly not sustainable in the future. The causal link between genetic damage and carcinogenicity is well documented; however, the limitations of genotoxicity and carcinogenicity testing assays, the presence of additional nongenotoxic mechanisms, issues of species-specific effects, and the lack of mechanistic insights provide enormous scientific challenges.

The genetic toxicology testing battery has been designed to be highly sensitive in predicting chemical carcinogenicity. The genetic toxicology testing battery detects 93% of carcinogens (Kirkland et al. 2005, 2006). However, the extremely high sensitivity of the testing paradigm comes at a cost of very low specificity. For instance, 50% of noncarcinogens among pharmaceuticals have some findings in genotoxicity assays (Hoffmann and Hartung 2006; MacDonald 2004; Snyder 2009; Snyder and Green 2001). Furthermore, the current testing paradigm for carcinogenicity fails to detect a variety of nongenotoxic carcinogens, such as hormone-type agents, which may impose a considerable human health risk.

The 2-year rodent carcinogenicity bioassays are associated with high costs, high animal burden, a long time frame (often ≥ 3 years), limited accuracy, and the uncertainty associated with extrapolating from rodents to humans. Additional frustrations exist because of the limited predictability of the 2-year bioassay and, in particular, the problem of predicting false positives. For instance, the Carcinogenic Potency Database (Gold 2010) includes results from chronic, long-term animal cancer tests with mice, rats, and hamsters amounting to a total of 6,540 individual experiments with 1,547 chemicals; 751 of those chemicals (51%) have positive findings in rodent studies. Similarly, when one considers all chronically used human pharmaceuticals, some 50% induce tumors in rodents, yet only 20 human pharmaceutical compounds have been identified as carcinogens in epidemiological studies, despite the fact that quite a large number of epidemiological studies have been carried out on these compounds (e.g., nonsteroidal antiinflammatory drugs, benzodiazepines, and phenobarbital). This high incidence of tumors in bioassays has led to questions concerning the human relevance of tumors induced in rodents (Knight et al. 2006; Ward 2008).

In addition, concerns have been raised regarding the age-related tumor incidence in the rodent bioassay, with many tumors arising only after 18–24 months of exposure. In contrast, most compounds designated by the International Agency for Research on Cancer (2010) to be human carcinogens induce tumors in rodents within 12 months of exposures (with some exceptions of tumors not arising until up to 18 months). A growing body of evidence indicates that rodent tumors (particularly from nongenotoxic chemicals) result from alternative or secondary mechanisms that are unique to rodents and therefore are not necessarily predictive of human hazard. Therefore, the dilemma remains as to the relevance of rodent bioassay findings to human mechanisms of carcinogenicity and human cancer risk.

In summary, dependency on the rodent model as a gold standard of cancer risk assessment neglects the high number of false positives and clearly has serious limitations. Consequently, there is a growing appeal for a paradigm change after “50 years of rats and mice.” Recent reports from the National Research Council of the U.S. National Academies have focused on the challenges of toxicology and human risk assessment, appealing for dramatic changes to move forward (National Research Council 2007a, 2007b). In addition, the current demands for toxicity and carcinogenicity testing of high-volume chemicals together with limitations of animal use as stipulated by the European Union’s Registration, Evaluation, Authorisation and Restriction of Chemicals (REACH) regulation (Combes et al. 2006) will require revolutionary changes in testing and risk assessment paradigms.

To develop a road map for this needed paradigm change in carcinogenicity testing, a workshop was held in August 2009 in Venice, Italy, titled “Genomics in Cancer Risk Assessment.” This workshop brought together toxicologists from academia and industry and government regulators and risk assessors from the United States and the European Union to discuss the state-of-the-art in developing alternative testing strategies for carcinogenicity, focusing on contributions from the omics technologies. What follows is a highlight of the major conclusions and suggestions from this workshop as a path forward.

## The Challenge: Addressing Human Relevance

Participants concluded, based on both scientific and technical reasons, that the current assays need to be improved if not replaced by mechanism-based assays that would enable assessment of relevance of observed findings for human health and disease. Preferentially, these assays should be derived from human *in vitro* cellular models and should be properly validated against human *in vivo* data (coming from investigations of patients, clinical trials, and human biomarker research) through translational research. New assays optimally should not rely on animal use and should have sufficient throughput that would satisfy demands for testing. Despite an array of currently established *in vitro* and *in vivo* testing methods, deriving insights into carcinogenic mechanisms with respect to the oncogenic potential of chemicals to humans is a difficult task. One suggestion to improve human cancer risk assessment is to incorporate genomic and genetic approaches into the risk assessment paradigm (Ellinger-Ziegelbauer et al. 2008a; Guyton et al. 2008). Because molecular or informational pathways are generally conserved across species from yeast to man (Ideker et al. 2001) and because pathways are generally well represented on all assay platforms, although the actual representation may vary, genomic-based systems biology approaches have the potential to bridge the *in vitro* and *in vivo* preclinical assays with human-relevant cancer mechanisms. Where pathways differ between species, those differences can provide valuable insight in understanding molecular mechanisms responsible for species-specific responses to exposures. Remarkable research activity is being conducted in the use of genomics as a tool for toxicological evaluation of chemicals and pharmaceuticals, including genotoxic and nongenotoxic carcinogenicity investigations after both *in vitro* and *in vivo* exposures. Several successful demonstrations of limited and well-defined applications of genomic approaches to risk assessment have included the identification of hazards through classification of test or unknown compounds with compounds of a particular class (Hamadeh et al. 2002; van Delft et al. 2004), the generation of mechanistic information through molecular pathway analysis revealing biological processes effected by exposures (Amundson et al. 2005; Aubrecht and Caba 2005; Heinloth et al. 2003; Liu et al. 2006), and the prediction of a limited number of specific potential adverse effects from exposures (Ellinger-Ziegelbauer et al. 2005, 2008b; Fielden et al. 2007; Heinloth et al. 2004; van Delft et al. 2004).

Recent work by Lamb and colleagues integrates gene expression transcriptomics data from human cells with chemical and drug information together with disease information into what the authors refer to as a “Connectivity Map” (Lamb 2007; Lamb et al. 2006). Applying this approach to systems biology, the authors demonstrate their ability to discover the activity of a compound (referred to as a “perturbagen”) in a molecular pathway that the compound had not previously been known to affect. Although this data set was clearly limited, the results were promising. The implication of this study is that a more robust database of information might provide insight into potential risks relevant to human exposures to a wide variety of chemicals and drugs.

For nongenotoxic chemicals, the important signals for identifying human cancer hazards may be detected in shorter-term studies using toxicogenomics approaches, rather than routinely relying on data from 2-year rodent bioassays. Characteristic pathway-associated gene expression signatures have been identified in the liver and kidneys of rats after short-term (2 weeks or 90 days) treatment with carcinogens, which fit very well the known mechanisms of carcinogenesis (Auerbach et al. 2010). These can discriminate genotoxic and nongenotoxic carcinogens from noncarcinogens, with a typical sensitivity of 0.92 and specificity of 0.88, a significant improvement over the performance of current animal-based models. This may contribute to weight-of-evidence considerations in cancer risk assessment. Pathway-associated gene signatures may serve as biomarkers that may then help to predict a carcinogenic hazard in different organs even after short-term treatment (Ellinger-Ziegelbauer et al. 2008a).

Perhaps the ideal approach for assessing human risk associated with exposure to chemical carcinogens would be an *in vitro* assay that would apply genomics in conjunction with bioinformatics methods to interrogate mechanisms of action of tested compounds. This information would then be used for the development and qualification of genomic biomarkers for chemical carcinogenesis that would provide the basis for mechanism-based risk assessment. *In vitro* classification of compounds for true genotoxic and nongenotoxic carcinogenicity, using cell lines, including human liver cellular models, can be 70–90% accurate (van Delft et al. 2004). Furthermore, developing a genomic-based biomarker approach to identify irrelevant findings, for instance, findings from *in vitro* chromosome damage assays that are false positives and irrelevant findings for human risk assessment, would have specific, immediate human relevance and would provide significant improvement in current genotoxicity testing (Aubrecht and Caba 2005; Goodsaid et al. 2010). Tissue-specific *in vitro* approaches to recognize carcinogens need to be developed, and such efforts would focus research appropriately on producing necessary tools to address organ specificity and ultimately improve prediction and risk assessment (carcinoGENOMICS 2007; Vinken et al. 2008). Furthermore, high-throughput approaches that are currently being evaluated by the U.S. Environmental Protection Agency (EPA) ToxCast effort and by the National Institute of Environmental Health Sciences, National Toxicology Program, the National Human Genome Research Institute, and the U.S. EPA Tox21 high-throughput screening effort have concentrated on a functional genomic approach that would allow triage of compounds for testing. These efforts could thus provide the opportunity to focus limited resources on the most important, problematic compounds (Dix et al. 2007).

## Gaps to Be Addressed

The ultimate, long-term goal for applying genomic-based approaches to human cancer risk assessment is the eventual replacement of the current testing paradigm, which includes genotoxicity and carcinogenicity testing, with mechanism-based assays that would allow both hazard detection and assessment of the relevance to humans, not rodents, of observed findings. For this to become a reality, molecular alterations and mechanistic insight derived from human cellular models need to be correlated with injury or potential for injury in humans. Linking outcomes of toxicogenomics investigations *in vitro* to ongoing human omics-based biomarker studies could help to make this happen. Consequently, more extensive data that are derived from human studies are needed, in particular, appropriate samples from individuals exposed at low but well-defined levels. Because development of biomarkers suitable to monitor exposure in human populations is essential to human risk and relevance, approaches to develop genomic biomarkers for individuals exposed to a specific agent of concern would provide the necessary advances to develop broad biomarker-based approaches (McHale et al. 2010). Genomic approaches have the potential to facilitate the discovery of surrogate biomarkers that are gene expression signatures or expression patterns of proteins or metabolites linked with a particular phenotype. This “phenotypic anchoring” of genomic signatures (Paules 2003) would allow for the use of patterns as surrogate biomarkers that may be useful in treatment and risk assessment decision making in the clinical or regulatory setting, even if the underlying molecular mechanism is not fully understood. This approach has been demonstrated powerfully with gene-expression–based surrogate biomarkers that have provided information to clinicians about the prognosis of breast tumors and that have helped in the design of appropriate therapeutic treatment regimes (Paik et al. 2004; Sotiriou and Pusztai 2009; van ’t Veer et al. 2002). Thus, genomic approaches that use appropriate human samples from well-designed studies of exposed human populations may yield powerful novel biomarkers useful both in the clinical setting and in risk assessment.

Systems toxicology approaches should also pay attention to the relative sensitivity of humans and the variability in the human response. As carcinogens are increasingly recognized to affect multiple molecular mechanisms and thus multiple cellular pathways, insights into these mechanisms could inform new predictive approaches, such as predictive *in vitro* assays, and allow for the development of specific, mechanism-based human biomarkers. These new biomarkers could then provide insight into the genetic variability in responses to the risk of developing cancers. The use of such mechanistic data will play a key role in the future of risk assessment to aid in identification of additional sources of human variability and susceptibility (e.g., background diseases and processes, coexposures) and improve prediction of interactions across environmental and endogenous exposures. Identifying mechanistic drivers of adverse responses will be particularly important in the risk assessment of exposures at low doses. Once progress is made in these areas, it may be possible to address the dose–response curve in an individual, which can take multiple forms, depending on such factors as the individual’s genetic background, the target tissue affected, and the actual internal dose of a specific compound or chemical. Linking outcomes of *in vitro* toxicogenomics investigations to ongoing human omics-based biomarker studies may make this happen. Goodsaid et al (2010) noted that regulators appear to be willing to accept such approaches where use is clearly defined, evidence is strong, and approaches are validated and qualified for regulatory use. In general, educating stakeholders is crucial to successfully implement the new testing paradigm.

Toxicogenomics applications require further technological standardization as well as biological standardization, especially with respect to the annotation of genes and pathways related to toxicologically relevant end points. Further progress must be made in systems toxicology applications, that is, developing integrative approaches across multiple genomic, genetic, molecular, and cellular assays to assess toxic events from a holistic perspective, as described with the Connectivity Map approach. The first generation toxicogenomics studies used microarray-based whole-genome analysis of gene expression modifications. Current technologies analyze the interplay between epigenetic events (e.g., whole-genome DNA methylation and histone acetylation, modifications of levels of mRNA, modifications of levels of regulatory microRNAs) and proteomic and metabolomic events, thus increasing the potential of identifying pivotal pathways whose perturbation functionally contributes ultimately in inducing toxicity and disease. To accomplish this will require better data analysis tools, specifically bioinformatics-based decision-supporting tools, to help not only the research scientists but also chemical and drug registrants and regulators. Furthermore, this will require publically accessible databases that integrate different methods and types of information, from emerging omics data types to traditional pathological, toxicological, physiological, molecular, and clinical data. New methods of training and familiarizing all parties involved with these new tools and strategies will be needed. This training may require new additions to existing curricula for students and special, targeted training opportunities for professionals.

A major challenge is the need to phenotypically anchor genomic responses from *in vitro* studies and testing assays on chemical carcinogenesis to human pathophysiology. The critical need for human relevancy is not a new problem but a serious issue that has appeared to be intractable in the past, because of, at least in part, the paucity of critical human samples and information. For this to be overcome now, cooperation and data sharing between private and public research partners and broad collaborative efforts will be required.

## The Path Forward

**Figure 1:**
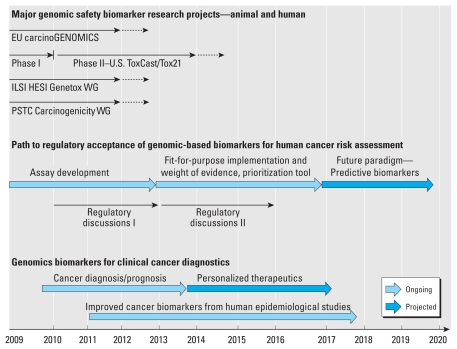
Road map for human cancer risk assessment. Four major projects that aim to develop a genomic biomarker approach for human cancer risk assessment have been initiated in the United States and the European Union (EU). carcinoGENOMICS (EU’s Sixth Framework Programme, funded from 2009 to 2012) attempts to develop human cell-based *in vitro* models and tools to detect carcinogens. The Phase I and the Phase II studies, conducted by the U.S. EPA (ToxCast program) and the National Institutes of Health (Tox21 program), exploit available biochemical and molecular assays to develop high-throughput, predictive assay panels. The International Life Sciences Institute Health and Environmental Sciences Institute (ILSI HESI) Genotoxicity Working Group (Genotox WG) focuses on developing an *in vitro* genomic biomarkers that can provide mechanistic context to *in vitro* genotoxicity testing. The aim of the FDA’s Critical Path Initiative Predictive Safety Testing Consortium Carcinogenicity Working Group (PSTC Carcinogenicity WG) is to develop an *in vivo* genomic biomarker detecting hepatocarcinogenicity, applicable for use in early drug development. These initiatives are expected to yield biomarkers and assays that initially could be applied as weight of evidence in a fit-for-purpose manner. This will provide the time and opportunity for refining the methods and assembling a new testing paradigm. Concurrently, progress in the development of cancer diagnostic methods is expected to provide data specific to human cancer that, after integration with *in vitro* and preclinical genomics data, will open additional avenues to further increase human relevance of this new testing paradigm. Solid lines indicate current funding activities; dotted lines indicate anticipated continuation of funding or projects into the future.

Toxicogenomics is considered a very promising but complex technology requiring coordinated planning and execution that is highly transparent and participatory to all segments of toxicology and risk assessment: academia, government, industry, nongovernmental organizations, and the public. Although we are still far from replacing the current testing paradigm for cancer risk assessment, toxicogenomics approaches at their current stage may be used to add weight of evidence to a risk assessment, by supporting additional studies in the presence or absence of a certain mechanism or mode of action and by describing end points that are not evaluated in a “checklist” manner but through an integrated scientific approach. Much work is needed, but a number of activities both in Europe and in the United States are already ongoing that will help address the issue of bringing genomic approaches into human cancer risk assessment (Figure 1). In support of this approach, the U.S. Food and Drug Administration in a joint undertaking with the European Union’s European Medicines Agency has already invited the pharmaceutical industry to submit omics data in the context of the registration of novel compounds (Goodsaid et al. 2010). Importantly, the REACH legislation states:

The Commission, Member States, industry and other stakeholders should continue to contribute to the promotion of alternative test methods on an international and national level including computer supported methodologies, *in vitro* methodologies, as appropriate, those based on toxicogenomics, and other relevant methodologies. (REACH Online 2006)

Regulatory agencies should not simply wait for these future developments but should anticipate that these novel toxicogenomics-based approaches also require the development of novel quality standards and further regulatory criteria. We feel it is critical to establish a formal global dialogue among regulatory agencies to coordinate and facilitate progress and acceptance of anticipated advances (Figure 1, Regulatory discussions, Phase I and II). In this line, we call upon international regulatory agencies and risk assessment decision makers to establish two phases of regular dialogues, each over a 2- or 3-year period, that will address initially the implementation of genomics-based assay development and then the use of genomics-based weight-of-evidence information in risk assessment decisions. With the progress being made in science today using genomics approaches, particularly using readily accessible human material, in conjunction with rigorous traditional scientific endeavors, we are now at a point when genomic applications in human studies can yield important information for better human cancer risk assessment.
